# Experience of Personal Loss Due to Drug Overdose Among US Adults

**DOI:** 10.1001/jamahealthforum.2024.1262

**Published:** 2024-05-31

**Authors:** Alene Kennedy-Hendricks, Catherine K. Ettman, Sarah E. Gollust, Sachini N. Bandara, Salma M. Abdalla, Brian C. Castrucci, Sandro Galea

**Affiliations:** 1 Department of Health Policy and Management, Johns Hopkins Bloomberg School of Public Health, Baltimore, Maryland; 2 Division of Health Policy and Management, University of Minnesota School of Public Health, Minneapolis; 3 Department of Mental Health, Johns Hopkins Bloomberg School of Public Health, Baltimore, Maryland; 4 Department of Epidemiology and Department of Global Health, Boston University School of Public Health, Boston, Massachusetts; 5 de Beaumont Foundation, Bethesda, Maryland; 6 Department of Epidemiology, Boston University School of Public Health, Boston, Massachusetts

## Abstract

**Importance:**

Since 1999, over 1 million people have died of a drug overdose in the US. However, little is known about the bereaved, meaning their family, friends, and acquaintances, and their views on the importance of addiction as a policy priority.

**Objectives:**

To quantify the scope of the drug overdose crisis in terms of personal overdose loss (ie, knowing someone who died of a drug overdose) and to assess the policy implications of this loss.

**Design, Setting, and Participants:**

This cross-sectional study used data from a nationally representative survey of US adults (age ≥18 years), the fourth wave of the COVID-19 and Life Stressors Impact on Mental Health and Well-Being (CLIMB) study, which was conducted from March to April 2023.

**Main Outcomes and Measures:**

Respondents reported whether they knew someone who died of a drug overdose and the nature of their relationship with the decedent(s). They also reported their political party affiliation and rated the importance of addiction as a policy issue. Logistic regression models estimated the associations between sociodemographic characteristics and political party affiliation and the probability of experiencing a personal overdose loss and between the experience of overdose loss and the perceived salience of addiction as a policy issue. Survey weights adjusted for sampling design and nonresponse.

**Results:**

Of the 7802 panelists invited to participate, 2479 completed the survey (31.8% response rate); 153 were excluded because they did not know whether they knew someone who died of a drug overdose, resulting in a final analytic sample of 2326 (51.4% female; mean [SD] age, 48.12 [0.48] years). Of these respondents, 32.0% (95% CI, 28.8%-34.3%) reported any personal overdose loss, translating to 82.7 million US adults. A total of 18.9% (95% CI, 17.1%-20.8%) of all respondents, translating to 48.9 million US adults, reported having a family member or close friend die of drug overdose. Personal overdose loss was more prevalent among groups with lower income (<$30 000: 39.9%; ≥$100 000: 26.0%). The experience of overdose loss did not differ across political party groups (Democrat: 29.0%; Republican: 33.0%; independent or none: 34.2%). Experiencing overdose loss was associated with a greater odds of viewing addiction as an extremely or very important policy issue (adjusted odds ratio, 1.37; 95% CI, 1.09-1.72) after adjustment for sociodemographic and geographic characteristics and political party affiliation.

**Conclusions and Relevance:**

This cross-sectional study found that 32% of US adults reported knowing someone who died of a drug overdose and that personal overdose loss was associated with greater odds of endorsing addiction as an important policy issue. The findings suggest that mobilization of this group may be an avenue to facilitate greater policy change.

## Key Points

**Question:**

What is the magnitude of personal overdose loss (ie, knowing someone who died of a drug overdose) in the US, and what are the policy implications of this loss?

**Findings:**

In this cross-sectional study of 2326 US adults, 32% reported knowing someone who died of a drug overdose. Experiencing personal overdose loss was associated with greater odds of endorsement of addiction as an important policy issue.

**Meaning:**

The findings suggest that mobilizing the large portion of the US population that has experienced drug overdose loss may be an avenue to facilitating greater policy change.

## Teaser

This cross-sectional study uses data from a nationally representative survey to evaluate the proportion of US adults who know someone who died of a drug overdose and whether this experience is associated with viewing addiction as an important policy issue.

## Introduction

Drug overdose continues to be one of the most severe and long-lasting public health crises in the US. Since 1999, more than 1 million people have died from a drug overdose, with overdose death totals reaching more than 100 000 annually in 2021 and 2022.^1,2^ Overdose is a contributor to the marked declines in life expectancy in the US, a trend beginning prior to the COVID-19 pandemic.^3^ Opioids are involved in most overdose deaths, with the proliferation of powerful synthetic opioids like fentanyl and polysubstance use accelerating the rising rate of overdose deaths in recent years.^2,4^

While the economic costs of the overdose crisis are estimated to exceed $1 trillion annually in the US,^5^ a more comprehensive accounting of the costs, particularly the effects of these deaths on loved ones and community members, is needed. Unexpected or sudden deaths of loved ones may lead to financial strain, lower productivity, weakened social ties, loneliness, and diminished health across the life course.^6,7^ However, limited research has explored the consequences of bereavement specifically due to drug overdose. One recent longitudinal study found that children with a parent who died of a drug overdose had increasing rates of mental health treatment in the years following this death.^8^ For family members or friends of the decedent, the experience of death from overdose may also be exacerbated by the stigmatized nature of overdose.^9,10^ Research on the effects of the overdose crisis on broader social networks and communities is limited in part by the methodologic challenges of distinguishing the causal effects of the overdose crisis from endogenous factors contributing to a community’s existing risk of addiction and overdose. Areas of the US that have been disproportionately affected by the overdose crisis face social and economic challenges^11,12,13^; while it may be likely that these challenges worsen in the face of heightened overdose rates, empirical evidence is lacking.

Capturing the magnitude of the overdose crisis through the lens of personal overdose loss (ie, knowing someone who died of a drug overdose) can also inform understanding of the politics of this issue.^14^ The loved ones of individuals affected by major health issues can form powerful political constituencies that organize, build interest groups, and advocate for policy change.^15^ Prior research on the politics of the overdose crisis has primarily focused on the relationship between community overdose rates and life expectancy declines and voting behavior.^16,17,18,19,20,21^ An underlying assumption of these studies was that the overdose crisis drove voters in highly affected areas rightward on the political spectrum in recent elections. However, a study using individual-level data found no association between perceptions of the local severity of the overdose crisis and voting behavior in the 2018 midterm elections.^20^ To our knowledge, missing from this body of research is data on whether the overdose crisis intersects with individuals’ political party identification. Although extreme political polarization currently exists, it is unknown whether the experience of personal overdose loss differs across political party groups and how this might affect perceived salience of drug overdose as a policy issue.

Limited research has quantified the number of US individuals affected by drug overdose in terms of personal overdose loss, the relational nature of that loss (eg, death of a family member, friend, or acquaintance), and the characteristics of those who have experienced a personal overdose loss. Recent data reported that 9% of US adults had a family member die of a drug overdose and that 16% had a family member who had experienced a nonfatal drug overdose.^22^ More fully characterizing this experience of personal overdose loss among US adults’ wider networks of family, friends, and acquaintances can reveal the broader scope of this crisis.

Using data from a nationally representative survey of US adults conducted in 2023, this study sought to quantify the scale of the drug overdose crisis in terms of personal overdose loss, the relational nature of that loss, and the characteristics of US adults affected by the experience of personal overdose loss, including political party affiliation. This study also considered the political implications of personal overdose loss by assessing whether this experience is associated with greater salience of overdose as a public policy priority and whether salience of this issue differs across political party groups.

## Methods

### Study Design, Population, and Setting

This cross-sectional study analyzed survey data from a nationally representative sample of US adults aged 18 years or older and followed the Strengthening the Reporting of Observational Studies in Epidemiology (STROBE) reporting guideline. The data were derived from the fourth wave of the COVID-19 and Life Stressors Impact on Mental Health and Well-Being (CLIMB) study,^23,24^ an ongoing longitudinal survey of a representative sample of US adults that began in March 2020 to assess the association between social and economic stressors and mental health and well-being. In the fourth wave, 2 new questions were added to the CLIMB survey instrument regarding respondents’ experience of personal overdose loss. CLIMB study participants were sampled from the NORC AmeriSpeak standing panel in March 2020, with a replenishment sample drawn from the AmeriSpeak panel in this fourth wave. The overall AmeriSpeak panel was constructed using probability-based methods from an address-based sampling frame encompassing 97% of US households; it is commonly used to derive nationally representative estimates in health-related survey research.^25,26,27^ The fourth wave of the CLIMB survey was fielded from March 28 to April 17, 2023. Of the 7802 panelists invited to participate in this wave, 2479 completed the survey (31.8% response rate). Panelists provided electronic informed consent before initiating the survey. eAppendix 1 in Supplement 1 describes the comparability of this sample with Current Population Survey benchmarks. This study was approved by the Johns Hopkins institutional review board.

### Measures

The key variable of interest was whether the respondent had any personal overdose loss. We adapted 2 questions from a 2015 Kaiser Health Tracking Poll^28^: “Do you personally know anyone who has died from a drug overdose?” Respondents could answer “yes,” “no,” or “don’t know.” We excluded respondents who answered “don’t know” from our analysis. Respondents who answered “yes” were then asked, “Who do you know that has died from a drug overdose?” Response options were nonexclusive and included “a family member,” “a close friend,” or “an acquaintance.” A secondary outcome of interest measured whether respondents viewed addiction as a public policy priority. Respondents were asked whether addressing drug addiction should be an extremely important priority, a very important priority, a somewhat important priority, or not an important priority to address. We dichotomized this measure, coding responses that addressing drug addiction should be an extremely important or very important priority as 1 and the remaining response options as 0.

Sociodemographic information encompassed measures collected by AmeriSpeak on all panelists, including age, sex, 9-level census division of residence, race and ethnicity, educational level, annual household income, homeowner status, and marital status. Race and ethnicity were self-reported and analyzed because they are important social categories associated with patterns of drug overdose risk. Categories included Hispanic, non-Hispanic Black, non-Hispanic White, and non-Hispanic other (those who identified as ≥2 racial identities or as Asian). The CLIMB survey also included questions regarding respondents’ current health insurance coverage and financial status relative to 1 year previously. The latter was measured using a question adapted from the Survey of Consumer Attitudes and Behavior,^29^ which asked, “Would you say that in comparison to a year ago, you and your family living in your household are financially better off, about the same, or worse off now?” We constructed a binary measure of financial well-being and coded respondents reporting being “worse off now” as 1 and respondents who were financially “better off” or “about the same” as 0. To capture political party affiliation, the CLIMB survey used a question modified from the Pew Research Center that asked, “Do you consider yourself a Democrat, a Republican, an independent, or none of these?” In regression models, we collapsed the response options “independent” and “none of these” into a single category.

### Statistical Analysis

First, we calculated the percentage of respondents who reported experiencing any personal overdose loss (regardless of the nature of the relationship) and calculated separately the percentages of respondents who reported having a family member or close friend vs an acquaintance who died of a drug overdose. We multiplied these percentages by the total number of US adults aged 18 years or older reported in the 2020 US census (n = 258 343 281) to estimate the numbers of US adults who had experienced a personal overdose loss. Pearson χ^2^ tests of differences in row proportions were used to assess unadjusted differences across sociodemographic, geographic, and political party groups in the experience of any personal overdose loss. Survey weights were used in all analyses to adjust for sampling design and nonresponse.

A logistic regression model estimated the association between the aforementioned sociodemographic and political characteristics and the odds of experiencing a personal overdose loss. We also used a logistic regression model to estimate the probability of viewing addiction as an extremely or very important priority as a function of the experience of personal overdose loss, sociodemographic characteristics, and political party affiliation. In a sensitivity analysis, we replaced any personal overdose loss with a variable capturing whether the respondent experienced the death specifically of a family member or close friend. To test whether the strength of the association between personal overdose loss and viewing addiction as an extremely or very important priority varied by political party affiliation, we interacted the 3-category measure of political party affiliation with the binary measure of experiencing a personal overdose loss. We then used postestimation margins to calculate the adjusted mean probabilities within each political party group by personal overdose loss status.

All statistical analyses were conducted using Stata SE, version 14.2 (StataCorp LLC) and used the Stata survey estimation feature to adjust for sampling design and nonresponse. Two-sided *P* < .05 was considered significant.

## Results

**Figure 1.**
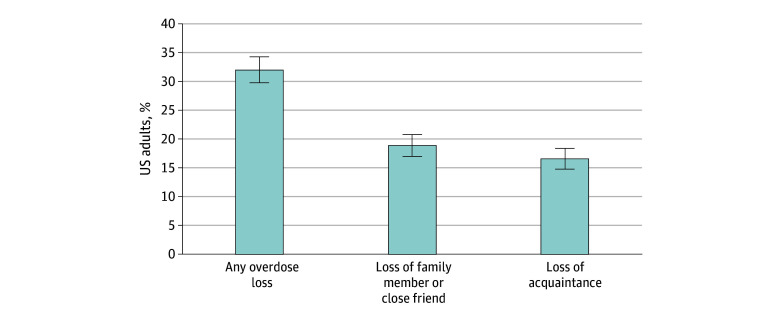
Experience of Personal Overdose Loss Among US Adults, 2023 Percentages were generated from responses among a nationally representative sample of US adults surveyed in 2023 and incorporate survey weights to adjust for nonresponse (n = 2326). Error bars represent 95% CIs.

Of 2479 individuals who completed the CLIMB survey, 153 were excluded because they did not know whether they knew someone who died of a drug overdose, resulting in a final analytic sample of 2326. Of these individuals, 52.4% were female and 48.6% were male; mean (SD) age was 48.12 (0.48) years. A total of 11.8% were Black; 17.0%, Hispanic; 62.1%, White; and 9.0%, other race. Across demographic benchmarks, this sample of US adults was similar to adults in the Current Population Survey data^30^ (eTable 1 in Supplement 1), providing evidence of the national representativeness of this sample. Of the respondents 32.0% (95% CI, 28.8%-34.3%) reported any personal overdose loss, translating to 82.7 million affected US adults (Figure 1). A total of 18.9% (95% CI, 17.1%-20.8%) of respondents, or 48.9 million US adults, reported that a family member or close friend died of a drug overdose, and 16.6% (95% CI, 14.9%-18.4%), or 42.8 million US adults, reported that an acquaintance died of a drug overdose.

**Figure 2.**
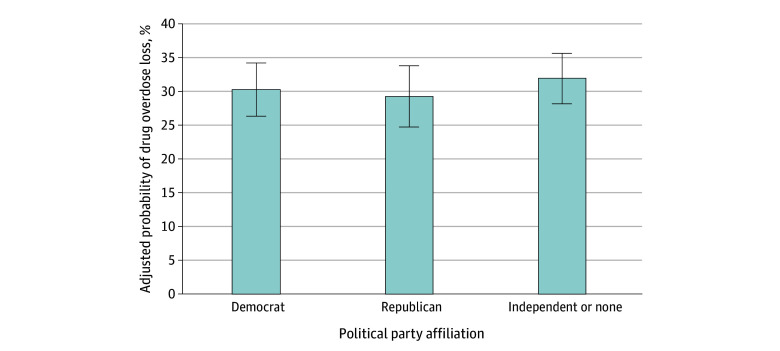
Adjusted Estimated Probability of Experiencing Personal Overdose Loss by Political Party Affiliation Group Model was adjusted for sociodemographic characteristics. The sample included 2287 individuals. Error bars represent 95% CIs.

The Table displays the unadjusted characteristics of US adults who had and had not experienced a personal overdose loss and the adjusted odds of experiencing personal overdose loss as a function of these characteristics. Personal overdose loss was most prevalent among adults aged 45 to 54 years (37.1%) and 55 to 64 years (36.7%); White adults (36.4%); households with annual income below $30 000 (39.9% vs 26.0% for income≥$100 000); individuals who were widowed, divorced, or separated (43.6%); Medicaid enrollees (51.3%); residents of nonmetropolitan areas (39.6%); and respondents reporting a worsening financial situation over the past year (41.3%). The experience of personal overdose loss was also most prevalent among respondents living in the New England region (44.5%). Notably, there were no differences in personal overdose loss by political party affiliation (Democrat: 29.0%; Republican: 33.0%; independent or none: 34.2%). Unadjusted differences by sex and educational level were not significant. These patterns were generally consistent in the regression model adjusting for covariates. No difference was found in the probability of experiencing personal overdose loss across political party groups even after adjusting for sociodemographic and geographic characteristics (Table and Figure 2).

**Table.** Characteristics of US Adults Who Reported Experiencing a Personal Overdose Loss and Association Between Characteristics and the Odds of Experiencing Personal Overdose Loss

Characteristic	Respondents, weighted %		*P* value^a^	Adjusted OR (95% CI)^b^
	No overdose loss	Overdose loss		
Age, y				
18-24	75.4	24.6	.05	1.14 (0.60-2.19)
25-34	70.5	29.5		1.37 (0.87-2.16)
35-44	66.4	33.6		1.76 (1.15-2.68)^c^
45-54	62.9	37.1		1.94 (1.25-3.00)^c^
55-64	63.2	36.8		1.88 (1.28-2.78)^c^
≥65	70.3	29.7		1 [Reference]
Sex				
Female	67.5	32.5	.67	0.93 (0.75-1.16)
Male	68.5	31.5		1 [Reference]
Race and ethnicity				
Hispanic	71.4	28.6	<.001	0.71 (0.51-0.98)^d^
Non-Hispanic Black	79.4	20.6		0.34 (0.22-0.53)^c^
Non-Hispanic White	63.6	36.4		1 [Reference]
Non-Hispanic other^e^	76.6	23.4		0.57 (0.37-0.88)^d^
Educational level				
Less than high school	66.7	33.3	.34	1.30 (0.72-2.35)
High school graduate or equivalent	66.7	33.3		0.99 (0.68-1.45)
Some college or associate’s degree	65.3	34.7		1.17 (0.85-1.60)
Bachelor’s degree	70.8	29.2		1.02 (0.73-1.42)
Postgraduate study or professional degree	71.7	28.3		1 [Reference]
Household income, $				
<30 000	60.1	39.9	<.001	1.23 (0.79-1.91)
30 000 to <60 000	69.7	30.3		0.89 (0.63-1.26)
60 000 to <100 000	65.5	34.5		1.34 (1.00-1.79)^d^
≥100 000	74.0	26.0		1 [Reference]
Marital status				
Married	69.7	30.3	<.001	1 [Reference]
Widowed, divorced, or separated	56.4	43.6		1.49 (1.12-1.99)^c^
Never married	72.3	27.7		0.87 (0.64-1.19)
Health insurance				
Commercial	72.2	27.8	<.001	1 [Reference]
Medicare	66.4	33.6		1.62 (1.12-2.32)^c^
Medicaid	48.7	51.3		2.59 (1.70-3.94)^c^
Other or uninsured	70.1	29.9		1.20 (0.78-1.83)
Home ownership status				
Does not own home	64.7	35.3	.05	1.25 (0.95-1.65)
Owns home	69.5	30.5		1 [Reference]
Geographic region				
New England	55.4	44.6	.04	2.40 (1.36-4.23)^c^
Mid-Atlantic	65.7	34.3		1.57 (0.96-2.55)
East North Central	65.1	34.9		1.63 (1.05-2.52)^d^
West North Central	75.3	24.7		1 [Reference]
South Atlantic	66.8	33.2		1.71 (1.11-2.64)^d^
East South Central	62.1	37.9		2.01 (1.10-3.68)^d^
West South Central	72.3	27.7		1.32 (0.78-2.23)
Mountain	71.4	28.6		1.17 (0.70-1.95)
Pacific	71.8	28.2		1.26 (0.78-2.03)
Metropolitan statistical area				
Nonmetropolitan area	60.4	39.6	<.006	1.17 (0.85-1.60)
Metropolitan area	69.2	30.8		1 [Reference]
Financial situation since previous year				
Worse off now	58.7	41.3	<.001	1.62 (1.28-2.05)^c^
About the same or better off	71.8	28.2		1 [Reference]
Political party affiliation				
Democrat	71.0	29.0	.13	1 [Reference]
Republican	67.0	33.0		0.95 (0.71-1.27)
Independent or none	65.8	34.2		1.08 (0.84-1.39)

Abbreviation: OR, odds ratio.

^a^ Bivariable analysis used Pearson χ^2^ tests of differences in row proportions of respondents with and without a personal overdose loss.

^b^ Estimated from logistic regression models. The fully adjusted model controlled for all variables displayed in the table.

^c^ *P* < .01.

^d^ *P* < .05.

^e^ Other included respondents identified in the AmeriSpeak panel as 2 or more races or as Asian.

**Figure 3.**
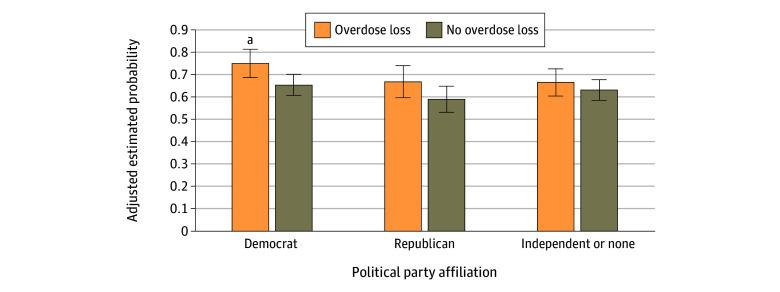
Estimated Probability of Viewing Addiction as an Extremely or Very Important Policy Priority by Personal Overdose Loss Experience and Political Party Affiliation Model was adjusted for sociodemographic characteristics. The sample included 817 Democrats, 562 Republicans, and 888 individuals with an independent or no affiliation. Error bars represent 95% CIs. ^a^Significant difference in viewing addiction as a very or extremely important policy priority among those who have or have not experienced personal overdose loss after adjusting for covariates.

The experience of any personal overdose loss was associated with greater odds of viewing addiction as an extremely or very important policy issue (adjusted odds ratio [AOR], 1.37; 95% CI, 1.09-1.72) after adjusting for sociodemographic and geographic characteristics and political party affiliation. Full regression model results are displayed in eTable 2 in Supplement 1. In a sensitivity analysis testing the association between experiencing the death of a family member or close friend from drug overdose and views on addiction as a policy priority, the AOR was directionally consistent but no longer significant (1.26; 95% CI, 0.96-1.66) (eTable 3 in Supplement 1). Figure 3 displays the differences in the adjusted mean probabilities of viewing addiction as a policy priority among those with and without any personal overdose loss across respondents affiliated with different political parties. While larger proportions of those who had experienced a personal overdose loss viewed addiction as a policy priority within all political party groups, after adjusting for covariates, only among Democrats was there a significant association between experiencing a personal overdose loss and viewing addiction as a policy priority. Full regression model results are shown in eTable 4 in Supplement 1. However, no significant differences were found in the strength of this association across political party groups in the model when formally testing for differences in a regression model interacting personal overdose loss with political party affiliation (eTable 5 in Supplement 1).

## Discussion

This cross-sectional study of data from a nationally representative survey of US adults found that an estimated 82.7 million US adults have known someone who died of a drug overdose, including 49 million who experienced the death of a family member or close friend. Notably, we found no difference in the experience of personal overdose loss by political party affiliation.

The findings suggest that drug overdose is an issue that affects a substantial proportion of US residents and that knowing someone who died of a drug overdose may be associated with moderately greater propensity to view addiction as a very or extremely important policy priority. That this large group of bereaved US adults, who are not as visible as other communities defined by less stigmatized health issues,^31^ reported this issue as a policy priority suggests the potential for this group to be more engaged in policy advocacy at the federal, state, or local level. There was no association between personal overdose loss of a family member or close friend and viewing addiction as a policy priority. On the one hand, we might expect individuals with more intimate relationships with individuals who died of drug overdose to feel more strongly about the need for policy change. On the other hand, research suggests having a personal relationship with an individual with substance use disorder does not always translate to lower levels of stigma.^32,33^ Family members and close friends may experience courtesy stigma,^34^ meaning public disapproval and discrimination due to their close association with members of a stigmatized group.^10,35,36^ To avoid or minimize courtesy stigma, some family members and close friends may distance themselves from the stigmatized issue, which could involve deemphasizing its importance as a policy issue. Nevertheless, efforts to strengthen support for policy change should consider the role of bereaved populations. Prior communication research has found that personal stories told from the perspective of an affected family member may increase receptiveness to public health–oriented drug policy.^37,38^ This group also may be an underrecognized audience for communication about potential policy strategies.

Despite no differences in the experience of personal overdose loss across political party groups, our findings suggested an association between experiencing any personal overdose loss and viewing addiction as an important policy issue among the subgroup of respondents identifying as Democrats but not among the subgroups of Republicans or independents. However, base levels of endorsement of addiction as an important policy issue were high (>60%) across all groups. Drug overdose prevention may be an issue that is more politically feasible to inspire action than other, more polarized health policy issues. However, despite bipartisan consensus on the importance of this issue, there may be less consensus on the specific policy solutions (eg, greater investment in treatment and harm reduction–oriented solutions vs more focus on supply reduction through law enforcement efforts) that would achieve broad support and be most effective at addressing the crisis.^39^

The impact of the overdose crisis has not been distributed evenly across the US population. Indicators of greater economic vulnerability, including Medicaid enrollment and increased financial strain over the past year, were associated with greater likelihood of experiencing a personal overdose loss. Certain geographic regions, such as New England and nonmetropolitan areas, were disproportionately affected as well. These patterns align with data on the individual- and area-level characteristics of individuals who died of drug overdose on some dimensions, notably economic vulnerability^40^ and geographic heterogeneity.^41^ However, they vary in other ways from more recent surveillance data, as the overdose crisis has evolved rapidly over time. For example, while our data showed that White respondents were more likely to report knowing someone who died of a drug overdose, drug overdose mortality rates have accelerated in recent years among non-Hispanic Black populations and are significantly elevated among American Indian and Alaska Native individuals,^40,42^ an important group that we were unable to identify in sufficient numbers in this study.

Despite the aforementioned patterns, findings from this study also showed that no community or sociodemographic group in the US had not experienced personal overdose loss. Although personal overdose loss was reported more frequently among groups with lower income, it was still a common experience in groups of all economic strata, including among 26.0% of persons with annual household income over $100 000. That this is a common, shared experience among sociodemographic groups in the US population may help to reduce stigma regarding overdose death, unite otherwise disconnected groups, and mobilize leaders to advance policy solutions.

### Limitations

The findings should be considered in the context of the study’s limitations. First, personal overdose loss may be underreported due to the stigma of overdose. One way in which this may occur is when respondents are unaware of the cause of death due to lack of disclosure by next-of-kin. A second limitation is that we did not ask respondents about the recency of their personal overdose loss(es). This may explain why some patterns in the experience of personal overdose loss diverged from the most recent national data on individuals who died of drug overdose, notably racial and ethnic inequalities.^40,42^ Understanding the impact of personal overdose loss among American Indian and Alaska Native individuals is of particular importance given the disproportionate burden of the overdose crisis in this population^42^; future survey-based research should oversample among this population to ensure a sufficient sample to generate representative estimates.

## Conclusions

This cross-sectional study found that 32% of US adults, or 82.7 million, had someone they know die of a drug overdose, and most of this group had experienced the death of a relative or close friend. This experience extended across the political spectrum, offering a potential avenue for increasing the mobilization of this group and the political feasibility of needed policy action to decrease overdose deaths.

## Notes

Supplement 1. **eTable 1.** Comparison of Study Sample to National Data **eTable 2.** Association Between Personal Overdose Loss and Sociodemographic Characteristics and the Probability of Viewing Addiction as an Extremely or Very Important Policy Issue **eTable 3.** Association Between Loss of Family Member or Close Friend to Drug Overdose and Sociodemographic Characteristics and the Probability of Viewing Addiction as an Extremely or Very Important Policy Issue **eTable 4.** Stratified Models Estimating the Relationship Between Personal Overdose Loss and Viewing Addiction as an Extremely or Very Important Policy Issue Separately by Political Party Affiliation **eTable 5.** Logistic Regression Model Estimating Whether Relationship Between Personal Overdose Loss and Viewing Addiction as an Extremely or Very Important Policy Issue Varies by Political Party Affiliation by Testing Significance of Interaction Terms

Supplement 2. Data Sharing Statement

## References

[1] Ahmad F, Cisewski J, Rossen J, Sutton P. Provisional drug overdose death counts. National Center for Health Statistics, Centers for Disease Control and Prevention. 2023. Accessed October 11, 2023. https://www.cdc.gov/nchs/nvss/vsrr/drug-overdose-data.htm

[2] Spencer MR, Miniño AM, Warner M. Drug overdose deaths in the United States, 2001-2021. National Center for Health Statistics, Centers for Disease Control and Prevention. 2022. Accessed October 1, 2023. https://www.cdc.gov/nchs/products/databriefs/db457.htm

[3] Dowell D, Arias E, Kochanek K, . Contribution of opioid-involved poisoning to the change in life expectancy in the United States, 2000-2015. JAMA. 2017;318(11):1065-1067. doi:10.1001/jama.2017.9308 28975295 PMC5818798

[4] Mattson CL, Tanz LJ, Quinn K, Kariisa M, Patel P, Davis NL. Trends and geographic patterns in drug and synthetic opioid overdose deaths—United States, 2013-2019. MMWR Morb Mortal Wkly Rep. 2021;70(6):202-207. doi:10.15585/mmwr.mm7006a4 33571180 PMC7877587

[5] Commission on Combating Synthetic Opioid Trafficking Final Report. Commission on Combating Synthetic Opioid Trafficking; 2022.

[6] Stroebe M, Schut H, Stroebe W. Health outcomes of bereavement. Lancet. 2007;370(9603):1960-1973. doi:10.1016/S0140-6736(07)61816-9 18068517

[7] Keyes KM, Pratt C, Galea S, McLaughlin KA, Koenen KC, Shear MK. The burden of loss: unexpected death of a loved one and psychiatric disorders across the life course in a national study. Am J Psychiatry. 2014;171(8):864-871. doi:10.1176/appi.ajp.2014.13081132 24832609 PMC4119479

[8] Hulsey EG, Li Y, Hacker K, Williams K, Collins K, Dalton E. Potential emerging risks among children following parental opioid-related overdose death. JAMA Pediatr. 2020;174(5):503-504. doi:10.1001/jamapediatrics.2020.0613 32282028 PMC7154958

[9] Valentine C, Bauld L, Walter T. Bereavement following substance misuse: a disenfranchised grief. J Death Dying. 2016;72(4). doi:10.1177/0030222815625174

[10] Titlestad KB, Lindeman SK, Lund H, Dyregrov K. How do family members experience drug death bereavement? a systematic review of the literature. Death Stud. 2021;45(7):508-521. doi:10.1080/07481187.2019.1649085 31390307

[11] Saloner B, McGinty EE, Beletsky L, . A public health strategy for the opioid crisis. Public Health Rep. 2018;133(suppl 1):24S-34S. doi:10.1177/0033354918793627 30426871 PMC6243441

[12] Patrick SW, Faherty LJ, Dick AW, Scott TA, Dudley J, Stein BD. Association among county-level economic factors, clinician supply, metropolitan or rural location, and neonatal abstinence syndrome. JAMA. 2019;321(4):385-393. doi:10.1001/jama.2018.20851 30694320 PMC6439754

[13] Monnat SM. Demographic and geographic variation in fatal drug overdoses in the United States, 1999-2020. Ann Am Acad Pol Soc Sci. 2022;703(1):50-78. doi:10.1177/00027162231154348 37366474 PMC10292656

[14] Barry CL, Saloner B. Using policy tools to improve population health—combating the U.S. opioid crisis. N Engl J Med. 2021;385(23):2113-2116. doi:10.1056/NEJMp2102323 34890503

[15] Oliver TR. The politics of public health policy. Annu Rev Public Health. 2006;27(104):195-233. doi:10.1146/annurev.publhealth.25.101802.123126 16533115

[16] Bor J. Diverging life expectancies and voting patterns in the 2016 US presidential election. Am J Public Health. 2017;107(10):1560-1562. doi:10.2105/AJPH.2017.303945 28817322 PMC5607673

[17] Bilal U, Knapp EA, Cooper RS. Swing voting in the 2016 presidential election in counties where midlife mortality has been rising in white non-Hispanic Americans. Soc Sci Med. 2018;197(197):33-38. doi:10.1016/j.socscimed.2017.11.050 29220706

[18] Wasfy JH, Healy EW, Cui J, Stewart C III. Relationship of public health with continued shifting of party voting in the United States. Soc Sci Med. 2020;252:112921. doi:10.1016/j.socscimed.2020.112921 32203851

[19] Goodwin JS, Kuo YF, Brown D, Juurlink D, Raji M. Association of chronic opioid use with presidential voting patterns in US counties in 2016. JAMA Netw Open. 2018;1(2):e180450. doi:10.1001/jamanetworkopen.2018.0450 30646079 PMC6324412

[20] Gollust SE, Haselswerdt J. A crisis in my community? local-level awareness of the opioid epidemic and political consequences. Soc Sci Med. 2021;291:114497. doi:10.1016/j.socscimed.2021.114497 34710820

[21] Curtis LH, Hoffman MN, Califf RM, Hammill BG. Life expectancy and voting patterns in the 2020 U.S. presidential election. SSM Popul Health. 2021;15:100840. doi:10.1016/j.ssmph.2021.100840 34169139 PMC8209240

[22] Sparks G, Montero A, Kirzinger A, Valdes I, Hamel L. KFF Tracking Poll July 2023: substance use crisis and accessing treatment. KFF. 2023. Accessed October 1, 2023. https://www.kff.org/other/poll-finding/kff-tracking-poll-july-2023-substance-use-crisis-and-accessing-treatment/

[23] Ettman CK, Abdalla SM, Cohen GH, Sampson L, Vivier PM, Galea S. Low assets and financial stressors associated with higher depression during COVID-19 in a nationally representative sample of US adults. J Epidemiol Community Health. 2020;75(6):501-508. doi:10.1136/jech-2020-215213 33277339 PMC7722349

[24] Ettman CK, Cohen GH, Abdalla SM, . Persistent depressive symptoms during COVID-19: a national, population-representative, longitudinal study of U.S. adults. Lancet Reg Health. 2022;5:100091. 10.1016/j.lana.2021.100091 PMC8488314 34635882

[25] Gupta RS, Warren CM, Smith BM, . Prevalence and severity of food allergies among US adults. JAMA Netw Open. 2019;2(1):e185630. doi:10.1001/jamanetworkopen.2018.5630 30646188 PMC6324316

[26] Goldfarb JL, Kreps S, Brownstein JS, Kriner DL. Beyond the first dose—COVID-19 vaccine follow-through and continued protective measures. N Engl J Med. 2021;385(2):101-103. doi:10.1056/NEJMp2104527 33909962

[27] McGinty EE, Presskreischer R, Anderson KE, Han H, Barry CL. Psychological distress and COVID-19–related stressors reported in a longitudinal cohort of US adults in April and July 2020. JAMA. 2020;324(24):2555-2557. doi:10.1001/jama.2020.21231 33226420 PMC7684524

[28] DiJulio B, Firth J, Hamel L, Brodie M. Kaiser Health Tracking Poll: November 2015. KFF. 2015. Accessed April 10, 2016. https://kff.org/health-reform/poll-finding/kaiser-health-tracking-poll-november-2015/

[29] University of Michigan Survey Research Center Economic Behavior Program. Survey of Consumer Attitudes and Behavior, April 2019. ICPSR. 2022. Accessed April 25, 2024. https://www.icpsr.umich.edu/web/ICPSR/studies/38398/versions/V1

[30] Flood S, King M, Rodgers R, . Current Population Survey: version 11.0. IPUMS CPS. 2023. Accessed April 25, 2024. https://cps.ipums.org/cps/

[31] Volkow ND. Stigma and the toll of addiction. N Engl J Med. 2020;382(14):1289-1290. doi:10.1056/NEJMp1917360 32242351

[32] Eisenberg D, Downs MF, Golberstein E. Effects of contact with treatment users on mental illness stigma: evidence from university roommate assignments. Soc Sci Med. 2012;75(6):1122-1127. doi:10.1016/j.socscimed.2012.05.007 22703886

[33] Kennedy-Hendricks A, Barry CL, Gollust SE, Ensminger ME, Chisolm MS, McGinty EE. Social stigma toward persons with prescription opioid use disorder: associations with public support for punitive and public health-oriented policies. Psychiatr Serv. 2017;68(5):462-469. https://psychiatryonline.org/doi/10.1176/appi.ps.201600056. doi:10.1176/appi.ps.201600056 28045350

[34] Tsai AC, Kiang MV, Barnett ML, . Stigma as a fundamental hindrance to the United States opioid overdose crisis response. PLoS Med. 2019;16(11):e1002969. doi:10.1371/journal.pmed.1002969 31770387 PMC6957118

[35] Liahaugen Flensburg O, Richert T, Väfors Fritz M. Parents of adult children with drug addiction dealing with shame and courtesy stigma. Drugs Educ Prev Policy. 2023;30(6):563-572. doi:10.1080/09687637.2022.2099249

[36] Bottomley JS, Campbell KW, Titlestad KB, Feigelman W, Rheingold AA. Predictors of stigma, guilt, and shame among adults bereaved by fatal overdose. Omega (Westport). Published online August 8, 2023. doi:10.1177/00302228231194208 37553120

[37] Bachhuber MA, McGinty EE, Kennedy-Hendricks A, Niederdeppe J, Barry CL. Messaging to increase public support for naloxone distribution policies in the United States: results from a randomized survey experiment. PLoS One. 2015;10(7):e0130050. doi:10.1371/journal.pone.0130050 26132859 PMC4488484

[38] McGinty EE, White SA, Sherman SG, Lee R, Kennedy-Hendricks A. Framing harm reduction as part of an integrated approach to reduce drug overdose: a randomized message testing experiment in a nationally representative sample of U.S. adults, 2022. Int J Drug Policy. 2023;118:104101. doi:10.1016/j.drugpo.2023.104101 37352766

[39] Weiss M, Zoorob M. Political frames of public health crises: discussing the opioid epidemic in the US Congress. Soc Sci Med. 2021;281:114087. doi:10.1016/j.socscimed.2021.114087 34102424

[40] Kariisa M, Davis NL, Kumar S, . Vital signs: drug overdose deaths, by selected sociodemographic and social determinants of health characteristics—25 states and the District of Columbia, 2019-2020. MMWR Morb Mortal Wkly Rep. 2022;71(29):940-947. doi:10.15585/mmwr.mm7129e2 35862289 PMC9310633

[41] National Center for Health Statistics, Centers for Disease Control and Prevnetions. Drug overdose mortality by state. 2022. Accessed September 1, 2023. https://www.cdc.gov/nchs/pressroom/sosmap/drug_poisoning_mortality/drug_poisoning.htm 37639452

[42] Friedman J, Beletsky L, Jordan A. Surging racial disparities in the U.S. overdose crisis. Am J Psychiatry. 2022;179(2):166-169. doi:10.1176/appi.ajp.2021.21040381 35105165 PMC8820266

