# Supplementary material for: Experience of Personal Loss Due to Drug Overdose Among US Adults
Source: JAMA Health Forum. 2024 May 31;5(5):e241262. doi: 10.1001/jamahealthforum.2024.1262 (PMC11143465; doi:10.1001/jamahealthforum.2024.1262)
Supplement: Supplement 1. — eTable 1. Comparison of Study Sample to National Data eTable 2. Association Between Personal Overdose Loss and Sociodemographic Characteristics and the Probability of Viewing Addiction as an Extremely or Very Important Policy Issue eTable 3. Association Between Loss of Family Member or Close Friend to Drug Overdose and Sociodemographic Characteristics and the Probability of Viewing Addiction as an Extremely or Very Important Policy Issue eTable 4. Stratified Models Estimating the Relationship Between Personal Overdose Loss and Viewing Addiction as an Extremely or Very Important Policy Issue Separately by Political Party Affiliation eTable 5. Logistic Regression Model Estimating Whether Relationship Between Personal Overdose Loss and Viewing Addiction as an Extremely or Very Important Policy Issue Varies by Political Party Affiliation by Testing Significance of Interaction Terms [file jamahealthforum-e241262-s001.pdf]

# Supplemental Online Content

Kennedy-Hendricks A, Ettman CK, Gollust S, et al. Experience of personal loss due to drug overdose among US adults. *JAMA Health Forum.* Published online May 31, 2024. doi:10.1001/jamahealthforum.2024.1262

**eTable 1.** Comparison of Study Sample to National Data

**eTable 2.** Association Between Personal Overdose Loss and Sociodemographic Characteristics and the Probability of Viewing Addiction as an Extremely or Very Important Policy Issue

**eTable 3.** Association Between Loss of Family Member or Close Friend to Drug Overdose and Sociodemographic Characteristics and the Probability of Viewing Addiction as an Extremely or Very Important Policy Issue

**eTable 4.** Stratified Models Estimating the Relationship Between Personal Overdose Loss and Viewing Addiction as an Extremely or Very Important Policy Issue Separately by Political Party Affiliation

**eTable 5.** Logistic Regression Model Estimating Whether Relationship Between Personal Overdose Loss and Viewing Addiction as an Extremely or Very Important Policy Issue Varies by Political Party Affiliation by Testing Significance of Interaction Terms

This supplemental material has been provided by the authors to give readers additional information about their work.

© 2024 Kennedy-Hendricks A et al. *JAMA Health Forum.*

**eTable 1. Comparison of Study Sample to National Data**

| Demographic Category | Subcategory | Unweighted (%) | Weighted (%) | Benchmark (%) |
|---|---|---|---|---|
| **Age** | 18 - 34 | 23.2 | 29.0 | 29.0 |
| | 35 - 49 | 27.8 | 24.4 | 24.4 |
| | 50 - 64 | 25.7 | 24.2 | 24.2 |
| | 65 Plus | 23.3 | 22.4 | 22.4 |
| **Race and Ethnicity** | Hispanic | 16.7 | 17.4 | 17.4 |
| | Non-Hispanic Asian/Pacific Islander | 2.8 | 4.5 | 6.8 |
| | Non-Hispanic Black | 10.5 | 12.1 | 12.1 |
| | Non-Hispanic Others | 3.6 | 4.5 | 2.3 |
| | Non-Hispanic White | 66.4 | 61.4 | 61.4 |
| **Education Status** | Less than High School | 5.3 | 9.0 | 9.0 |
| | High School Equivalent | 17.5 | 29.0 | 29.0 |
| | Some College/Associate's Degree | 39.8 | 26.4 | 26.4 |
| | Bachelor's Degree or Higher | 20.9 | 19.9 | 22.5 |
| | Graduate Degree | 16.5 | 15.7 | 13.1 |
| **Sex** | Male | 49.9 | 48.8 | 48.8 |
| | Female | 50.1 | 51.2 | 51.2 |

Source: NORC at the University of Chicago. Project methods and transparency report. May 26, 2023. Benchmarks from the Current Population Survey.

© 2024 Kennedy-Hendricks A et al. *JAMA Health Forum.*

**eTable 2. Association Between Personal Overdose Loss and Sociodemographic Characteristics and the Probability of Viewing Addiction as an Extremely or Very Important Policy Issue**

| | Adjusted OR | |
|---|---|---|
| | Not Adjusted for Political Party | Adjusting for Political Party |
| Any Personal Overdose Loss | 1.36** (1.08, 1.71) | 1.37** (1.09, 1.72) |
| Age Category (Ref: ≥65 years) | | |
| 18-24 | 0.71 (0.39, 1.31) | 0.73 (0.40, 1.33) |
| 25-34 | 0.52** (0.34, 0.81) | 0.54** (0.34, 0.83) |
| 35-44 | 0.61* (0.40, 0.91) | 0.62* (0.41, 0.93) |
| 45-54 | 0.83 (0.55, 1.27) | 0.86 (0.56, 1.30) |
| 55-64 | 0.67* (0.46, 0.98) | 0.69 (0.47, 1.02) |
| Female (Ref: Male) | 1.24* (1.00, 1.53) | 1.21 (0.98, 1.50) |
| Race and Ethnicity (Ref: Not Hispanic [NH] White) | | |
| NH Black | 1.36 (0.92, 2.00) | 1.25 (0.84, 1.85) |
| NH Other | 1.25 (0.82, 1.91) | 1.22 (0.80, 1.87) |
| Hispanic | 1.06 (0.78, 1.43) | 1.02 (0.75, 1.39) |
| Education Level (Ref: Post-Graduate or Professional degree) | | |
| Less than High School | 1.61 (0.88, 2.92) | 1.68 (0.92, 3.07) |
| High School Graduate or Equivalent | 1.10 (0.77, 1.57) | 1.14 (0.80, 1.64) |
| Some College/Associate’s Degree | 0.97 (0.72, 1.31) | 1.00 (0.74, 1.35) |
| Bachelor’s Degree | 0.90 (0.66, 1.23) | 0.92 (0.67, 1.26) |
| Household Income (Ref: $100,000 or more) | | |
| Less than $30,000 | 0.70 (0.47, 1.04) | 0.72 (0.48,1.07) |
| $30,000 to under $60,000 | 1.23 (0.89, 1.70) | 1.27 (0.92, 1.77) |
| $60,000 to under $100,000 | 1.13 | 1.13 |

© 2024 Kennedy-Hendricks A et al. *JAMA Health Forum.*

| | | |
|---|---|---|
| | (0.86, 1.49) | (0.86, 1.49) |
| Marital Status (Ref: Married) | | |
| Widowed, Separated, or Divorced | 1.20<br>(0.89, 1.61) | 1.17<br>(0.88, 1.57) |
| Never Married | 0.99<br>(0.74, 1.32) | 0.94<br>(0.70, 1.26) |
| Insurance Status (Ref: Commercial) | | |
| Medicare | 1.14<br>(0.80, 1.63) | 1.13<br>(0.79, 1.62) |
| Medicaid | 0.92<br>(0.61, 1.40) | 0.91<br>(0.60, 1.38) |
| Other or Uninsured | 0.94<br>(0.63, 1.39) | 0.95<br>(0.64, 1.42) |
| Does Not Own Home (Ref: Owns home) | 1.54**<br>(1.19, 1.99) | 1.52**<br>(1.17, 1.97) |
| Geographic Region (Ref: West) | | |
| Northeast | 1.26<br>(0.89, 1.79) | 1.26<br>(0.89, 1.78) |
| Midwest | 1.08<br>(0.80, 1.45) | 1.11<br>(0.82, 1.49) |
| South | 1.36*<br>(1.03, 1.79) | 1.37*<br>(1.04, 1.81) |
| Lives in Non-Metro Area (Ref: Lives in Metro Area) | 1.02<br>(0.75, 1.40) | 1.02<br>(0.75, 1.40) |
| Worse Off Financially Than Last Year (Ref: Same or Better) | 0.85<br>(0.68, 1.07) | 0.88<br>(0.70, 1.11) |
| Political Party Affiliation (Ref: Democrat) | | |
| Republican | | 0.73*<br>(0.55, 0.97) |
| Independent or None | | 0.82<br>(0.64, 1.05) |
| Constant | 1.40<br>(0.88, 2.23) | 1.60<br>(0.98, 2.60) |
| Observations | 2,274 | 2,267 |

Adjusted odds ratios estimated from logistic regression models.
Two asterisks(**) indicate p-value<0.01; One asterisk(*) indicates p-value<0.05
Any personal drug overdose loss defined as reporting that they know someone who has died of a drug overdose.

© 2024 Kennedy-Hendricks A et al. *JAMA Health Forum.*

**eTable 3. Association Between Loss of Family Member or Close Friend to Drug Overdose and Sociodemographic Characteristics and the Probability of Viewing Addiction as an Extremely or Very Important Policy Issue**

| | Adjusted OR | |
|---|---|---|
| | Not Adjusted for Political Party | Adjusting for Political Party |
| Loss of Family Member or Close Friend to Drug Overdose | 1.24 (0.94 - 1.63) | 1.26 (0.96 - 1.66) |
| Age Category (Ref: ≥65 years) | | |
| 18-24 | 0.72 (0.39 - 1.32) | 0.73 (0.40 - 1.34) |
| 25-34 | 0.53** (0.34 - 0.82) | 0.54** (0.35 - 0.84) |
| 35-44 | 0.62* (0.41 - 0.93) | 0.63* (0.42 - 0.94) |
| 45-54 | 0.85 (0.56 - 1.29) | 0.87 (0.57 - 1.33) |
| 55-64 | 0.69 (0.47 - 1.01) | 0.71 (0.48 - 1.04) |
| Female (Ref: Male) | 1.24 (1.00 - 1.53) | 1.21 (0.98 - 1.50) |
| Race and Ethnicity (Ref: Not Hispanic [NH] White) | | |
| Hispanic | 1.04 (0.77 - 1.41) | 1.01 (0.74 - 1.37) |
| NH Black | 1.31 (0.89 - 1.93) | 1.20 (0.81 - 1.79) |
| NH Other | 1.21 (0.79 - 1.86) | 1.18 (0.77 - 1.81) |
| Education Level (Ref: Post-Graduate or Professional degree) | | |
| Less than High School | 1.58 (0.87 - 2.88) | 1.66 (0.91 - 3.02) |
| High School Graduate or Equivalent | 1.08 (0.76 - 1.53) | 1.12 (0.78 - 1.60) |
| Some College/Associate’s Degree | 0.96 (0.71 - 1.29) | 0.99 (0.73 - 1.33) |
| Bachelor’s Degree | 0.90 (0.66 - 1.22) | 0.91 (0.67 - 1.25) |
| Household Income (Ref: $100,000 or more) | | |
| Less than $30,000 | 0.70 (0.47 - 1.05) | 0.72 (0.48 - 1.08) |
| $30,000 to under $60,000 | 1.23 (0.89 - 1.71) | 1.28 (0.93 - 1.77) |
| $60,000 to under $100,000 | 1.14 (0.87 - 1.50) | 1.14 (0.87 - 1.50) |

© 2024 Kennedy-Hendricks A et al. *JAMA Health Forum.*

| | | |
|---|---|---|
| Marital Status (Ref: Married) | | |
| Widowed, Separated, or Divorced | 1.22<br>(0.91 - 1.63) | 1.19<br>(0.89 - 1.59) |
| Never Married | 0.98<br>(0.73 - 1.31) | 0.93<br>(0.69 - 1.25) |
| Insurance Status (Ref: Commercial) | | |
| Medicare | 1.15<br>(0.80 - 1.65) | 1.14<br>(0.79 - 1.64) |
| Medicaid | 0.95<br>(0.62 - 1.43) | 0.93<br>(0.61 - 1.41) |
| Other or Uninsured | 0.93<br>(0.63 - 1.38) | 0.95<br>(0.64 - 1.41) |
| Does Not Own Home (Ref: Owns home) | 1.54**<br>(1.19 - 2.00) | 1.52**<br>(1.18 - 1.98) |
| Geographic Region (Ref: West) | | |
| Northeast | 1.29<br>(0.91 - 1.83) | 1.29<br>(0.91 - 1.83) |
| Midwest | 1.09<br>(0.81 - 1.46) | 1.12<br>(0.83 - 1.50) |
| South | 1.37*<br>(1.04 - 1.80) | 1.38*<br>(1.05 - 1.82) |
| Lives in Non-Metro Area (Ref: Lives in Metro Area) | 1.04<br>(0.76 - 1.42) | 1.04<br>(0.76 - 1.42) |
| Worse Off Financially Than Last Year (Ref: Same or Better) | 0.87<br>(0.69 - 1.10) | 0.90<br>(0.71 - 1.13) |
| Political Party Affiliation (Ref: Democrat) | | |
| Republican | | 0.72*<br>(0.54 - 0.96) |
| Independent or None | | 0.82<br>(0.64 - 1.06) |
| Constant | 1.45<br>(0.91 - 2.31) | 1.66*<br>(1.02 - 2.70) |
| Observations | 2,272 | 2,265 |

Adjusted odds ratios estimated from logistic regression models.
Two asterisks(**) indicate p-value<0.01; One asterisk(*) indicates p-value<0.05

© 2024 Kennedy-Hendricks A et al. *JAMA Health Forum.*

**eTable 4. Stratified Models Estimating the Relationship Between Personal Overdose Loss and Viewing Addiction as an Extremely or Very Important Policy Issue Separately by Political Party Affiliation**

| | Democrats n=817 | Republicans N=562 | Independents or None N=888 |
|---|---|---|---|
| Any Personal Overdose Loss | 1.62* (1.04 - 2.51) | 1.32 (0.86 - 2.05) | 1.13 (0.79 - 1.61) |
| Age Category (Ref: ≥65 years) | | | |
| 18-24 | 1.57 (0.52 - 4.77) | 0.97 (0.29 - 3.30) | 0.37* (0.15 - 0.93) |
| 25-34 | 0.58 (0.26 - 1.30) | 0.59 (0.24 - 1.47) | 0.41* (0.21 - 0.81) |
| 35-44 | 0.64 (0.31 - 1.31) | 0.85 (0.37 - 1.94) | 0.53 (0.28 - 1.01) |
| 45-54 | 1.09 (0.50 - 2.38) | 0.92 (0.43 - 1.96) | 0.63 (0.32 - 1.24) |
| 55-64 | 0.75 (0.39 - 1.45) | 0.75 (0.36 - 1.58) | 0.66 (0.35 - 1.23) |
| Female (Ref: Male) | 1.25 (0.86 - 1.80) | 1.07 (0.69 - 1.66) | 1.20 (0.85 - 1.70) |
| Race and Ethnicity (Ref: Not Hispanic [NH] White) | | | |
| Hispanic | 0.87 (0.51 - 1.50) | 0.79 (0.43 - 1.45) | 1.44 (0.90 - 2.33) |
| NH Black | 1.47 (0.88 - 2.48) | 1.65 (0.39 - 6.98) | 1.27 (0.65 - 2.47) |
| NH Other | 1.22 (0.59 - 2.50) | 0.57 (0.19 - 1.74) | 1.75 (0.94 - 3.26) |
| Education Level (Ref: Post-Graduate or Professional degree) | | | |
| Less than High School | 0.90 (0.31 - 2.62) | 2.52 (0.76 - 8.38) | 2.33 (0.93 - 5.86) |
| High School Graduate or Equivalent | 1.07 (0.57 - 2.00) | 1.17 (0.54 - 2.55) | 1.13 (0.64 - 2.00) |
| Some College/Associate's Degree | 1.00 (0.61 - 1.64) | 1.09 (0.55 - 2.16) | 1.04 (0.63 - 1.69) |
| Bachelor's Degree | 1.02 (0.62 - 1.69) | 0.85 (0.41 - 1.76) | 0.96 (0.58 - 1.61) |
| Household Income (Ref: $100,000 or more) | | | |
| Less than $30,000 | 0.73 (0.37 - 1.43) | 0.82 (0.35 - 1.94) | 0.63 (0.33 - 1.18) |
| $30,000 to under $60,000 | 1.29 (0.74 - 2.24) | 1.44 (0.78 - 2.66) | 1.15 (0.67 - 1.95) |
| $60,000 to under $100,000 | 1.49 | 0.92 | 1.09 |

© 2024 Kennedy-Hendricks A et al. *JAMA Health Forum.*

| | | | |
|---|---|---|---|
| | (0.92 - 2.41) | (0.53 - 1.60) | (0.70 - 1.71) |
| Marital Status (Ref: Married) | | | |
| Widowed, Separated, or Divorced | 1.03<br>(0.62 - 1.73) | 1.96*<br>(1.07 - 3.62) | 1.01<br>(0.65 - 1.59) |
| Never Married | 0.98<br>(0.61 - 1.58) | 0.83<br>(0.41 - 1.71) | 0.93<br>(0.59 - 1.47) |
| Insurance Status (Ref: Commercial) | | | |
| Medicare | 1.28<br>(0.68 - 2.39) | 1.20<br>(0.61 - 2.36) | 1.17<br>(0.65 - 2.09) |
| Medicaid | 0.95<br>(0.48 - 1.88) | 1.02<br>(0.41 - 2.53) | 0.92<br>(0.49 - 1.72) |
| Other or Uninsured | 0.90<br>(0.41 - 1.98) | 0.69<br>(0.33 - 1.42) | 1.17<br>(0.65 - 2.13) |
| Does Not Own Home (Ref: Owns home) | 1.46<br>(0.96 - 2.23) | 1.30<br>(0.70 - 2.41) | 1.70**<br>(1.14 - 2.53) |
| Geographic Region (Ref: West) | | | |
| Northeast | 1.23<br>(0.69 - 2.21) | 1.65<br>(0.80 - 3.44) | 1.14<br>(0.63 - 2.05) |
| Midwest | 1.23<br>(0.74 - 2.06) | 1.23<br>(0.68 - 2.22) | 1.00<br>(0.61 - 1.65) |
| South | 1.14<br>(0.70 - 1.85) | 2.62**<br>(1.49 - 4.60) | 1.11<br>(0.72 - 1.71) |
| Lives in Non-Metro Area (Ref: Lives in Metro Area) | 0.58*<br>(0.34 - 0.97) | 1.27<br>(0.75 - 2.15) | 1.45<br>(0.86 - 2.44) |
| Worse Off Financially Than Last Year (Ref: Same or Better) | 0.91<br>(0.58 - 1.44) | 0.62*<br>(0.40 - 0.95) | 1.11<br>(0.76 - 1.60) |
| Constant | 1.36<br>(0.61 - 3.01) | 0.94<br>(0.34 - 2.58) | 1.56<br>(0.75 - 3.21) |

© 2024 Kennedy-Hendricks A et al. *JAMA Health Forum.*

**eTable 5. Logistic Regression Model Estimating Whether Relationship Between Personal Overdose Loss and Viewing Addiction as an Extremely or Very Important Policy Issue Varies by Political Party Affiliation by Testing Significance of Interaction Terms**

| | Log Odds Regression Coefficient (95% CI) |
|---|---|
| Any Personal Overdose Loss | 0.48* (0.07 - 0.89) |
| Political Party Affiliation (Ref: Democrat) | |
| Republican | -0.29 (-0.63 - 0.05) |
| Independent or None | -0.11 (-0.41 - 0.19) |
| Personal Overdose Loss X Political Party Affiliation | |
| Overdose Loss X Republican | -0.13 (-0.70 - 0.45) |
| Overdose Loss X Independent/None | -0.32 (-0.85 - 0.21) |
| Age Category (Ref: ≥65 years) | |
| 18-24 | -0.33 (-0.93 - 0.28) |
| 25-34 | -0.62** (-1.06 - -0.18) |
| 35-44 | -0.48* (-0.89 - -0.07) |
| 45-54 | -0.16 (-0.58 - 0.26) |
| 55-64 | -0.37 (-0.75 - 0.02) |
| Female (Ref: Male) | 0.19 (-0.03 - 0.40) |
| Race and Ethnicity (Ref: Not Hispanic [NH] White) | |
| Hispanic | 0.02 (-0.28 - 0.33) |
| NH Black | 0.21 (-0.19 - 0.61) |
| NH Other | 0.20 (-0.23 - 0.62) |
| Education Level (Ref: Post-Graduate or Professional degree) | |
| Less than High School | 0.54 (-0.06 - 1.14) |
| High School Graduate or Equivalent | 0.14 (-0.22 - 0.50) |
| Some College/Associate's Degree | -0.00 |

© 2024 Kennedy-Hendricks A et al. *JAMA Health Forum.*

| | |
|---|---|
| | (-0.31 - 0.30) |
| Bachelor's Degree | -0.08<br>(-0.40 - 0.23) |
| Household Income (Ref: $100,000 or more) | |
| Less than $30,000 | -0.35<br>(-0.75 - 0.05) |
| $30,000 to under $60,000 | 0.24<br>(-0.09 - 0.56) |
| $60,000 to under $100,000 | 0.12<br>(-0.15 - 0.39) |
| Marital Status (Ref: Married) | |
| Widowed, Separated, or Divorced | 0.17<br>(-0.13 - 0.46) |
| Never Married | -0.06<br>(-0.35 - 0.24) |
| Insurance Status (Ref: Commercial) | |
| Medicare | 0.13<br>(-0.23 - 0.49) |
| Medicaid | -0.10<br>(-0.52 - 0.32) |
| Other or Uninsured | -0.05<br>(-0.44 - 0.35) |
| Does Not Own Home (Ref: Owns home) | 0.42**<br>(0.16 - 0.68) |
| Geographic Region (Ref: West) | |
| Northeast | 0.23<br>(-0.12 - 0.58) |
| Midwest | 0.10<br>(-0.19 - 0.40) |
| South | 0.32*<br>(0.04 - 0.60) |
| Lives in Non-Metro Area (Ref: Lives in Metro Area) | 0.03<br>(-0.29 - 0.34) |
| Worse Off Financially Than Last Year (Ref: Same or Better) | -0.13<br>(-0.36 - 0.10) |
| Constant | 0.43<br>(-0.07 - 0.92) |
| Observations | 2,267 |

Coefficients represent log odds.
Two asterisks(**) indicate p-value<0.01; One asterisk(*) indicates p-value<0.05
To test whether the relationship between personal drug overdose loss and viewing addiction as a policy priority (outcome) varied by political party affiliation, the model above interacted the overdose loss and political party affiliation variables.

© 2024 Kennedy-Hendricks A et al. *JAMA Health Forum.*
