# Supplementary material for: Experience of Personal Loss Due to Drug Overdose Among US Adults
Source: JAMA Health Forum. 2024 May 31;5(5):e241262. doi: 10.1001/jamahealthforum.2024.1262 (PMC11143465; doi:10.1001/jamahealthforum.2024.1262)
Supplement: Supplement 2. — Data Sharing Statement [file jamahealthforum-e241262-s002.pdf]

Data Sharing Statement

Kennedy-Hendricks. Experience of Personal Loss Due to Drug Overdose Among US Adults. *JAMA Health Forum*. Published May 31, 2024. doi:10.1001/jamahealthforum.2024.1262

Data
Data available: Yes
Data types: Deidentified participant data
How to access data: Data are available upon reasonable request for research purposes sent to CKE: cettman1@jhu.edu.
When available: With publication

Supporting Documents
Document types: None

Additional Information
Who can access the data: Researchers whose proposed use of the data has been approved.
Types of analyses: For specific research analyses approved by study team.
Mechanisms of data availability: After approval of a proposal and signed data access agreement.
